# Oral Selective TLR8 Agonist Selgantolimod Induces Multiple Immune Cell Responses in Humans

**DOI:** 10.3390/v13122400

**Published:** 2021-11-30

**Authors:** Natarajan Ayithan, Alip Ghosh, Ankit Dwivedi, Jeffrey J. Wallin, Susanna K. Tan, Diana Chen, Shyam Kottilil, Bhawna Poonia

**Affiliations:** 1 Division of Clinical Care and Research, Institute of Human Virology, University of Maryland School of Medicine, Baltimore, MD 21201, USA; nayithan@ihv.umaryland.edu (N.A.); AlipGhosh@ihv.umaryland.edu (A.G.); SKottilil@ihv.umaryland.edu (S.K.); 2 Institute for Genome Sciences, University of Maryland School of Medicine, Baltimore, MD 21201, USA; adwivedi@som.umaryland.edu; 3 Gilead Sciences Inc., Foster City, CA 94404, USA; jeffrey.wallin@gilead.com (J.J.W.); susanna.tan3@gilead.com (S.K.T.); diana.chen8@gilead.com (D.C.)

**Keywords:** toll-like receptor 8 (TLR8), chronic hepatitis B, mucosal-associated invariant T (MAIT), activation marker, pro-inflammatory cytokine, microarray, selgantolimod (SLGN)

## Abstract

TLR8 agonists have the potential for use as immunomodulatory components in therapeutic modalities for viral infections such as chronic HBV (CHB) and HIV. In this study, using peripheral blood samples from a phase 1a clinical trial, we examined the acute effects of a single oral administration of a selective TLR8 agonist on immune cell phenotypes. Administration of the TLR8 agonist selgantolimod (SLGN) in healthy individuals resulted in alteration in frequencies of peripheral blood monocytes, pDCs, mDCs and MAIT cells. Frequencies of mDCs and lymphoid cells significantly reduced after 8 h of SLGN administration, whereas pDC frequencies significantly increased, with changes possibly reflecting migration of different cell types between peripheral and tissue compartments in response to the agonist. Myeloid cell activation was evident by an upregulated expression of co-stimulatory molecules CD40 and CD86 accompanied by the production of IL-6 and IL-18 from these cells. Concomitantly, there was induction of the early activation marker CD69 on innate and adaptive lymphoid cells, including MAIT and NK cell subsets. Further, these activated lymphoid cells had enhanced expression of the effector molecules granzyme B and perforin. Microarray analysis of isolated lymphocytes and monocytes from baseline and post-SLGN treatment revealed changes in expression of genes involved in cellular response to cytokine stimulus, innate immune response, myeloid cell differentiation and antigen receptor-mediated signaling pathway. In a preliminary analysis of samples from CHB patients treated with selgantolimod, activation of innate and adaptive lymphocytes was evident. In conclusion, this first in-human study shows that selgantolimod administration in humans results in activation of multiple immune cell responses with antiviral potential.

## 1. Introduction

Chronicity of viral infections is manifested as defects in host innate and adaptive immune responses; thus, strategies to reinvigorate these responses are under evaluation as therapeutics. Novel synthetic compounds and small molecule agonists are being evaluated for clinical use as vaccine adjuvants and for therapeutic immunomodulation to achieve functional cure of viral infections. The promising immunomodulatory approaches that aim to stimulate an innate and/or adaptive immune system, including cytokines, therapeutic vaccines, strategies to block checkpoint molecules such as PD-1, CTLA-4, LAG3, activators of RIG-1 and Toll-like receptor (TLR) agonists.

Toll-like receptors are a family of pattern-recognition receptors that mediate innate and adaptive immune responses. TLR8 is located on the endosomal membranes of immune cells, including monocytes, myeloid DCs and regulatory T cells and recognizes pathogen-associated single-stranded RNA molecules [1,2,3,4,5,6]. TLR8 activation leads to secretion of pro-inflammatory cytokines, chemokines and type I interferons that initiate innate and adaptive immune responses. Preclinical and clinical studies have shown that downstream inflammatory immune mediators from TLR8 signaling result in preliminary efficacy for the treatment of cancer and viral infections [7]. Recently, we reported that in CHB patients, TLR8 signaling upregulated the follicular helper T (T_FH_) cell functions and rescued the exhausted phenotypes in an IL-12-dependent manner [8].

Preceding evidence from in vitro studies show that triggering of TLR8 with ssRNA40 induces pro-inflammatory cytokines IL-12 and IL-18 in hepatic monocytes, which further induce robust production of IFNγ by liver-resident CD161^Bright^ MAIT (mucosal-associated invariant T) and CD56^Bright^ NK cells [9]. Such activation of NK and MAIT cells via TLR8-mediated IL-12 and IL-18 production can support their antiviral function [10,11]. In addition, HIV-1 derived ssRNA40 triggered the activation of NK cells and neutrophils and induced pro-inflammatory cytokines such as IL-6, TNFα, IL-12p40, IL-18 and IFNγ; however, the cross-talk interaction and activation was required between monocytes/mDCs and NK, neutrophils for the enhancement of anti-HIV immunity [12,13,14]. Selgantolimod (SLGN), previously known as GS-9688, is a novel and potent selective small-molecule TLR8 agonist developed for potential use in chronic HBV therapy [15]. SLGN oral treatment improved HBsAg-specific B cell responses by enhancing the follicular helper T (T_FH_) cell functions via IL-12-dependent manner in a subset of CHB patients [8]. In a woodchuck model of CHB, SLGN administration induced pro-inflammatory cytokines IL-12p40, IL-18, IL-6, IL-1RA as well as antiviral cytokines TNFα and IFNγ and had an antiviral effect [16]. Phase 1 pharmacokinetic studies demonstrated rapid absorption and dose-dependent PK and PD activity of SLGN in healthy donors [17] and in CHB patients [18]. In vitro SLGN treatment activated effector cells, including antigen-specific CD8^+^ T cells, MAIT and NK cells in peripheral blood samples from CHB patients [19].

In this study, we aimed to identify the effects of SLGN oral administration on peripheral immune phenotypes and functions in healthy subjects. We report multi-faceted activation of monocytes and lymphocytes with a single SLGN dose, which indicates the immune modulatory potential of a TLR8 agonist. These readouts can serve as biomarkers of TLR8 pathway activity in future studies.

## 2. Materials and Methods

### 2.1. Patients and Samples

The phase 1a randomized, blinded, placebo-controlled study was conducted by Gilead Sciences, and details have been published previously [17]. Healthy subjects received a single dose of selgantolimod, and frozen peripheral blood mononuclear cells (PBMCs) from this trial were tested at the Institute of Human Virology, University of Maryland. Paired PBMC samples from baseline (BL) and 8 h post-selgantolimod administration time points from cohorts receiving 1.5 mg or 3 mg single doses were used throughout this study for subsequent experimental evaluation. For the Phase 1b study, PBMC from CHB patients that received 3 mg SLGN [18] were tested here.

### 2.2. Flow Cytometry (FACS) and Intracellular Cytokine Staining (ICS)

To investigate immunophenotyping by flow cytometry [8], PBMCs from BL and 8 h post-selgantolimod treatment were thawed, and after washing, 0.5–1 × 10^6^ cells/panel were labeled with antibodies (flow panels listed in Table S1) at 4 °C for 30 min. After staining, cells were washed twice with FACS buffer (1× PBS/2% FBS), followed by fixation with 2% paraformaldehyde. For intracellular staining, after staining with surface markers cells were fixed and permeabilized using a BD Cytofix/Cytoperm™ Fixation/Permeabilization kit for 20 min., followed by staining with intracellular antibodies at 4 °C for 30 min. Cells (approximately 200,000 events) were acquired on a BD FACS Aria III (Becton, Dickinson Company, Franklin Lakes, NJ, USA). Data analysis was performed with FlowJo software (Tree Star, San Carlos, CA, USA). Heatmap analysis for the change in the frequency of the activation markers and pro-inflammatory cytokines at 8 h compared to baseline on different subsets of immune cells among samples from the placebo (*n* = 5), 1.5 mg SLGN (*n* = 9) and 3.0 mg SLGN (*n* = 12) treatment groups were analyzed in R (version 4.0.3) using the heatmap.2() function.

### 2.3. Cell sorting, Total RNA Isolation and Microarray Analysis

RNA was isolated from sorted monocytes and lymphocytes using the miRNeasy mini kit (Qiagen) using manufacturer protocols. Briefly, PBMCs (10 × 10^6^) were first stained with live/dead aqua (ThermoFisher Scientific, Waltham, MA USA 02451) in PBS for 15 min. at dark in room temperature (RT), followed by staining with CD56-BV510 and CD14-APC monoclonal antibodies diluted in FACS buffer at 4 °C for 30 min. After washing, cells were resuspended in FACS buffer and filtered using nylon membrane screw capped falcon tubes. Cell sorting was carried out using a BD FACS Aria III machine. Lymphocytes and monocytes were sorted based on FSC and SSC, followed by CD14 exclusion from lymphocytes and CD56 exclusion from monocytes. Sorted cells were directly collected into 1 mL of sterile FBS in 5 mL FACS tube followed by centrifugation at 2500× *g* for 15 min. Pellets were resuspended in 0.5 mL RNAlater (Qiagen stabilization reagent) and stored in a −80 °C freezer until further analysis. Microarray analysis was performed using an Affymetrix gene expression microarray system. Data analysis was performed in R (version 4.0.3). The plot() function was used to generate volcano plots, and the hclust() and heatmap.2() functions were used to generate dendrograms and heatmaps, respectively, with the top 50% deregulated (high variance) genes. Mean gene expression values for all the probe-sets were compared between the baseline and 8 h post-treatment groups by a Student’s *t*-test. Only genes that were significantly differentially expressed (*p* ≤ 0.05, *t*-test) and changed at least 2-fold in monocytes and 1.5-fold in lymphocytes between baseline and 8 h post-treatment were considered for analysis. FDR adjustment was performed, but it failed to identify any significant probe at padj < 0.1.

### 2.4. Functional Enrichment Analysis

To describe the functional annotation and enrichment associated with the deregulated genes identified in monocytes and lymphocytes, the enrichment of functionally-grouped networks of Biological Process category Gene Ontology (GO) terms were analyzed using the ClueGO v2.5.8 [20] plugin of Cytoscape v3.9.0. The network was created with nodes as the most significant GO term representative of the functional group and edges as the functional association of different GO terms based on kappa score. The Homo sapiens reference genome in ClueGO was used as the reference background. To test the significance of enrichment, a right-sided hypergeometric statistical test was used, and the Benjamini–Hochberg method was used to correct for *p* values. The protein–protein interaction network, based on co-expression evidence among deregulated genes identified in monocytes and lymphocytes, was investigated using STRING v11.5 [21]. The interaction network was created with nodes as the gene/protein and edges as significant evidence from publicly available co-expression data accessible to STRING. All the edges with a medium confidence score ≥ 0.4 were represented on the network.

### 2.5. Statistical Analysis

Statistical analysis was performed in GraphPad prism software using a Wilcoxon matched-pairs signed-rank test for all datasets. Differences between the baseline and post-Selgantolimod time point were considered statistically significant at * *p* ≤ 0.05, ** *p* ≤ 0.01, *** *p* ≤ 0.001, **** *p* ≤ 0.0001 and ns (no significance).

## 3. Results

### 3.1. Modulation of Peripheral Blood Immune Cell Frequencies with SLGN Treatment in Healthy Subjects

**Figure 1:**
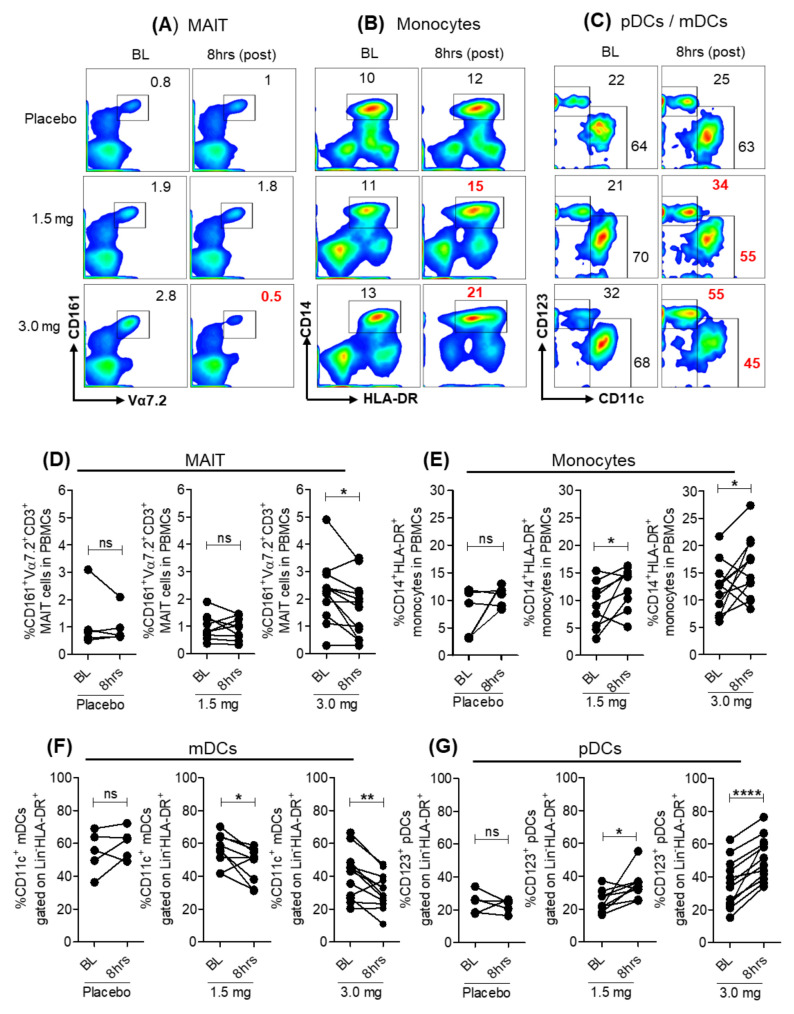
Frequencies of peripheral blood immune cells after Selgantolimod (SLGN) oral administration in healthy subjects. Flow cytometric analysis of PBMCs from BL (Pre) and 8 h (post-treatment) from individuals administered placebo (*n* = 5) or indicated dosage of SLGN (1.5 mg (*n* = 9) and 3.0 mg (*n* = 12)). Representative figures show gating for (**A**) MAIT (CD161^+^Vα7.2^+^), (**B**) CD14^+^HLA-DR^+^ monocytes and (**C**) CD123^+^ pDCs and CD11c^+^ mDCs. Comparative analyses of frequencies of immune cell subsets from baseline (BL) and 8 h samples in placebo or SLGN treated individuals (1.5 or 3.0 mg) for (**D**) MAIT, (**E**) monocytes, (**F**) mDCs and (**G**) pDCs are shown. Significance calculated by a Wilcoxon matched-pairs signed-rank test. *p* values ≤ 0.05 *, 0.01 ** and 0.0001 **** show grades of significance. ns: no significance.

We evaluated the effect of oral SLGN administration on the frequencies of myeloid and lymphoid cells by flow cytometry. Among lymphocytes, a significant reduction in the frequency of vα7.2^+^CD161^+^ MAIT cells 8 h after SLGN administration was evident (Figure 1A,D). CD3^+^, CD8^+^ T lymphocytes and total CD56^+^ NK cells were reduced, but differences did not reach statistical significance (Figure S1A–C). The frequencies of CD14^+^HLA-DR^+^ monocytes (Figure 1B,E and Figure S1D–F) and CD123^+^ plasmacytoid DCs (pDCs) gated on Lin^−^HLA-DR^+^CD11c^−^ were significantly increased at 8 h post-SLGN treatment compared to BL in both 1.5 mg and 3.0 mg dose groups but not in placebo controls. Conversely, a significant reduction in frequencies of CD11c^+^ myeloid DCs (mDCs) gated on Lin^−^HLA-DR^+^CD123^−^ was observed in 8 h post-SLGN treatment samples (Figure 1C,F,G).

These data show that a single dose of SLGN alters frequencies of monocytes and lymphocytes in peripheral blood of healthy individuals, possibly due to re-distribution of these cells between blood and tissue compartments.

### 3.2. SLGN Activated Myeloid and Lymphoid Immune Cells in Peripheral Blood

**Figure 2:**
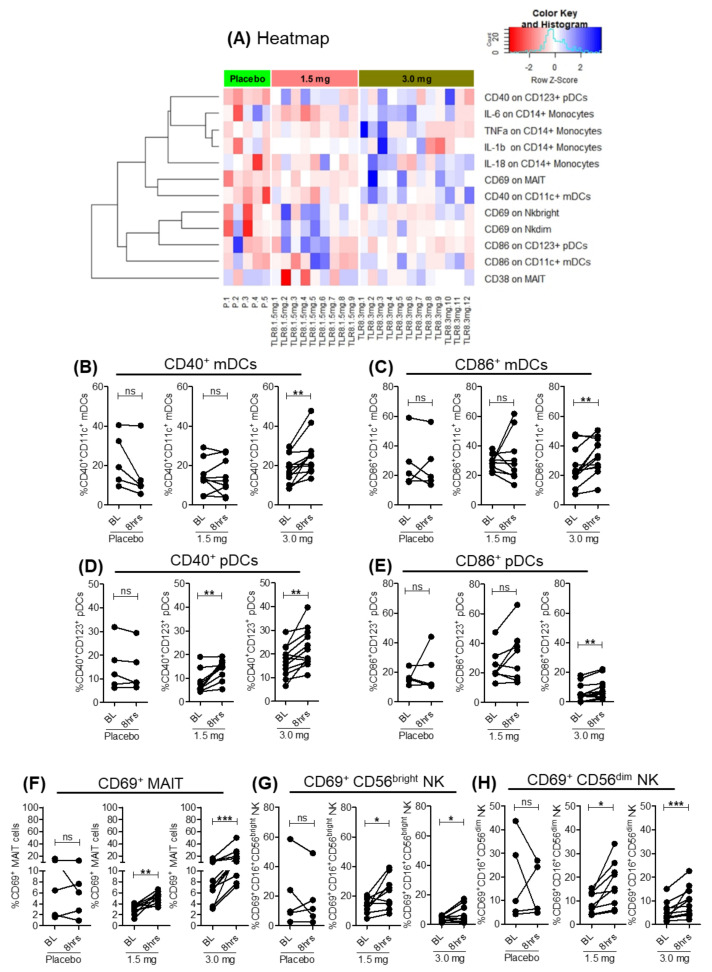
Activation of myeloid and lymphoid cells after oral-SLGN treatment in healthy subjects. (**A**) Heatmap representation of the activation markers (CD38, CD69, CD40 and CD86) and pro-inflammatory cytokines (IL-6, IL-18, TNFα and IL-1β) on different subsets of immune cells. Change in the frequency of the activation markers and cytokines at 8 h compared to baseline among samples from the placebo (*n* = 5), 1.5 mg SLGN (*n* = 9) and 3.0 mg SLGN (*n* = 12) treatment groups were presented in the heatmap. Frequency of activation markers are represented for (**B**) CD40^+^, (**C**) CD86^+^ on mDCs and (**D**) CD40^+^ and (**E**) CD86^+^ on pDCs, respectively, from baseline and 8 h samples in placebo, 1.5 or 3.0 mg SLGN-treated individuals. Comparative analyses of frequencies of (**F**) CD69^+^ on MAIT, (**G**) CD16^+^CD56^bright^ and (**H**) CD16^+^CD56^dim^ on NK cell subsets are shown. Significance was calculated by a Wilcoxon matched-pairs signed-rank test, and *p* value ≤ 0.05 *, 0.01 ** and 0.001 *** show grades of significance. ns: no significance.

Next, we examined the effects of SLGN on the activation of myeloid and lymphoid cells. The 3.0 mg SLGN dose significantly increased activation of monocytic populations, as evident by elevated expression of co-stimulatory markers CD40 and CD86 on mDCs (Figure 2A–C and Figure S2A) and pDCs (Figure 2A,D,E and Figure S2B), whereas 1.5 mg had a milder effect with only CD40 upregulated on pDCs (Figure 2A,D). Among lymphocytes, significant cell surface upregulation of the early activation marker CD69 was present on MAIT and CD56^+^ NK cell subsets (CD16^+^CD56^bright^ and CD16^+^CD56^dim^) after 8 h post-SLGN treatment, whereas no change was observed in CD38 activation on MAIT (Figure 2A,F–H and Figure S2C–E). Thus, a single oral dose of SLGN resulted in early activation of myeloid and lymphoid cells in healthy subjects.

### 3.3. Pro-Inflammatory Cytokines Induced in Monocytes with Selgantolimod

**Figure 3:**
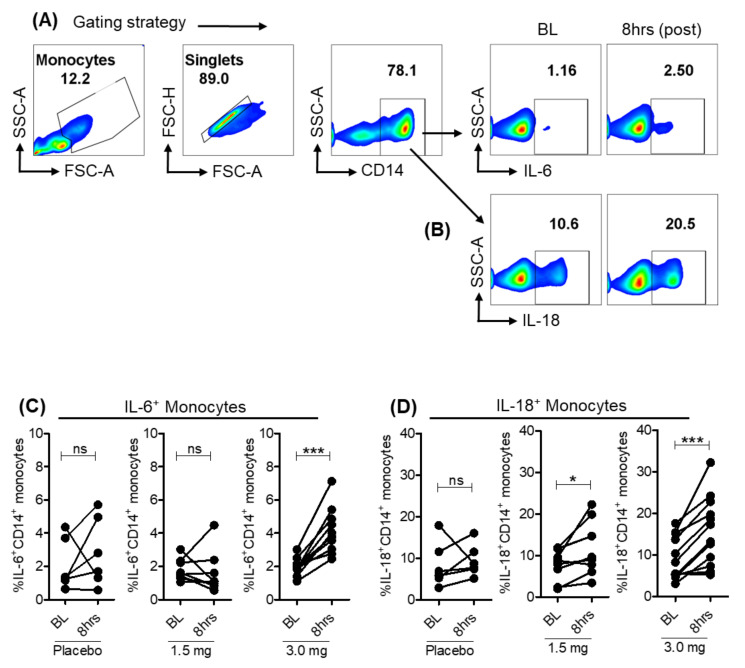
Pro-inflammatory cytokines in monocytes from SLGN-treated individuals. Pseudo color flow plots show gating strategy for measuring ex vivo expression of pro-inflammatory cytokines (**A**) IL-6 and (**B**) IL-18 in CD14^+^ monocytes. Frequencies of cytokine producing monocytes from baseline and 8-h samples in placebo, 1.5 or 3.0 mg SLGN-treated individuals are presented for (**C**) IL-6 and (**D**) IL-18, respectively. Significance was calculated by a Wilcoxon matched-pairs signed-rank test, and *p* value ≤ 0.05 * and 0.001 *** show grades of significance. ns: no significance.

TLR8 agonists induce a range of inflammatory mediators including antiviral cytokines, chemokines and interferons. Pharmacodynamic evaluation of SLGN previously showed a dose-dependent increase in pro-inflammatory and antiviral cytokines IL-12p40, IL-1RA, TNFα and IFNγ in serum samples from healthy subjects [17]. To investigate the cell types producing these cytokines, we tested cytokine production ex vivo using intracellular cytokine staining. As expected, SLGN increased the frequency of CD14^+^ monocytes producing the pro-inflammatory cytokines IL-6^+^ (Figure 2A and Figure 3A,C) and IL-18^+^ (Figure 2A and Figure 3B,D) with moderate or no significant induction of TNFα^+^ and IL-1β^+^ (Figure 2A). These data reveal that a selective-TLR8 agonist activates myeloid cells, resulting in production of pro-inflammatory cytokines in these cells.

### 3.4. Selgantolimod Treatment Enhanced Production of Granzyme B and Perforin in MAIT and NK Cells

**Figure 4:**
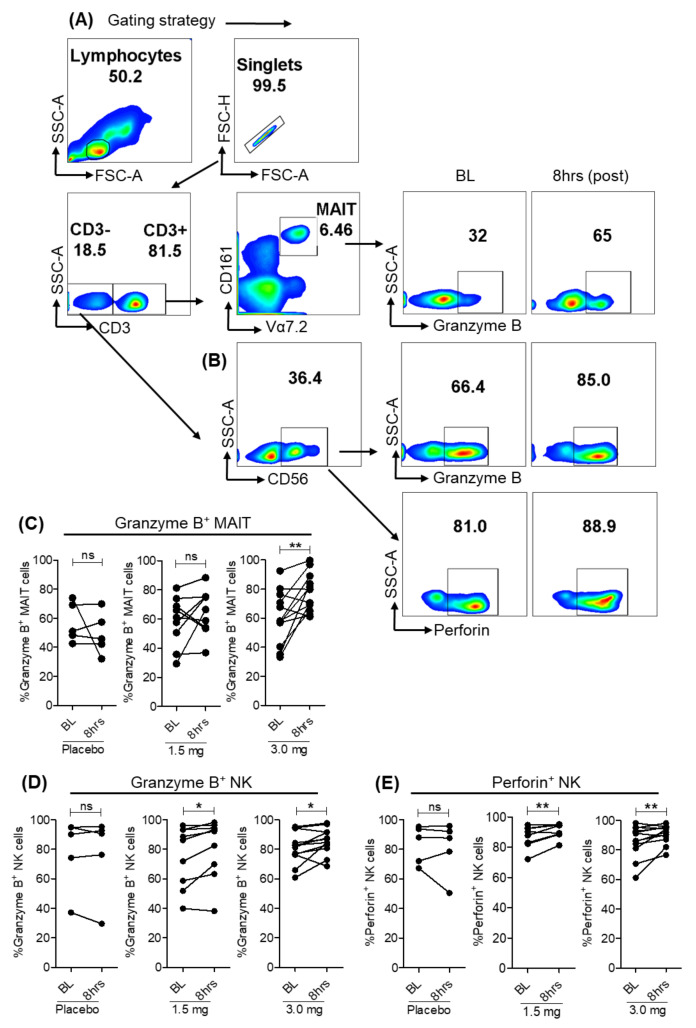
Effector molecules granzyme B and perforin after SLGN treatment. (**A**,**B**) Representative flow plots show gating strategy to measure ex vivo expression of Granzyme B in MAIT and Granzyme B and perforin in NK cells in response to 3.0 mg SLGN treatment. Relative frequencies are shown for (**C**) Granzyme B on MAIT cells, (**D**) Granzyme B and (**E**) Perforin on CD56^+^ NK cells. Significance was calculated by a Wilcoxon matched-pairs signed-rank test, and *p* value ≤ 0.05 * and 0.01 ** show grades of significance. ns: no significance.

In vitro studies with human PBMCs showed that IL-18 induced via TLR8 pathway results in the activation of CD161^++^CD8^+^ MAIT, NK and NKT cells, resulting in production of antiviral effector molecules granzyme B, TNFα and IFNγ [22,23]. To test in vivo effects of TLR8 agonism, we compared baseline and 8 h post-SLGN dosed individuals. An increase in production of granzyme B in MAIT cells gated on CD3^+^ T cells, granzyme B and perforin in CD56^+^ NK cells occurred with agonist treatment (Figure 4A–E). It was previously reported that production of granzyme B and perforin by MAIT and NK cells requires a strong activation by the stimulation of IL-12/IL-18 in CHB patients [24]. Our data shows that oral administration of specific-TLR8 agonist enhanced the production of effector molecules by innate and adaptive lymphoid cells, which can possibly be explored for their direct antiviral effect during viral infections.

### 3.5. Activation of Lymphoid Cells after Oral Selgantolimod Treatment in CHB Subjects

**Figure 5:**
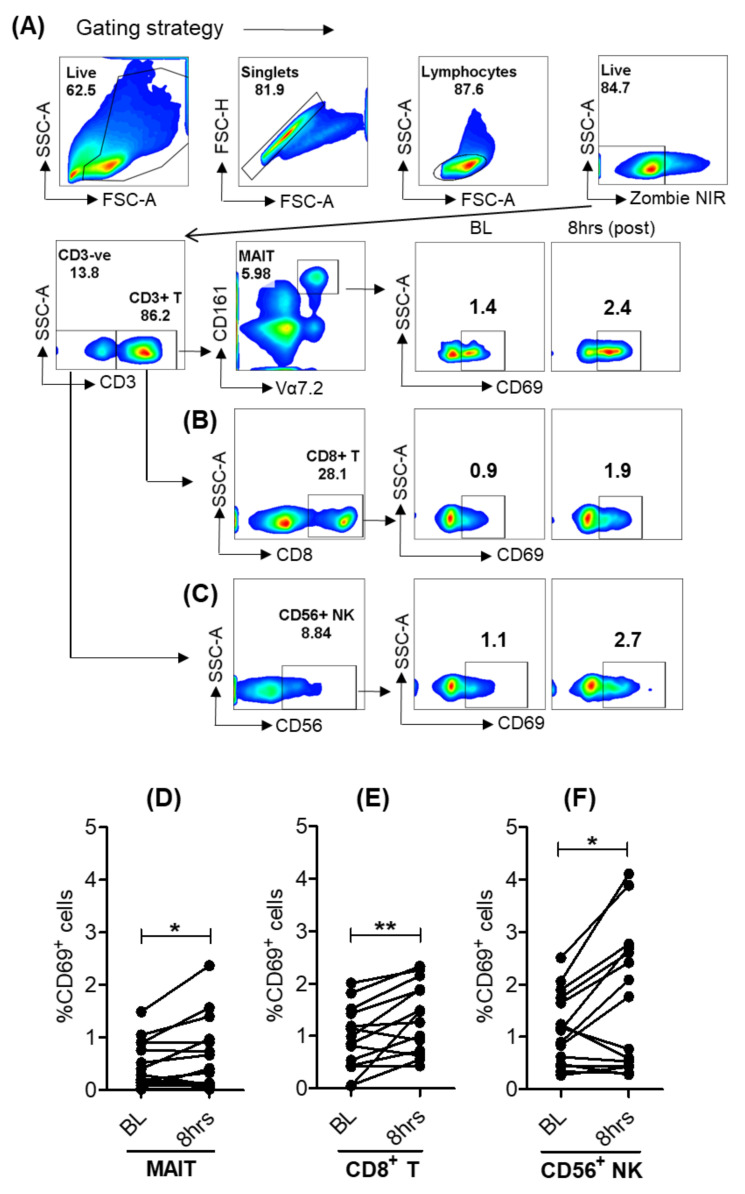
Activation of lymphoid cells after oral-SLGN treatment in CHB subjects. Gating strategy and cell surface expression of CD69 on (**A**) MAIT, (**B**) CD8^+^ T and (**C**) CD56^+^ NK cells. Comparative analyses of frequencies of CD69 on (**D**) MAIT, (**E**) CD8^+^ T and (**F**) CD56^+^ NK from baseline and 8 h samples in SLGN-treated (3.0 mg dose, *n* = 14) CHB patients. Significance was calculated by a two-tailed paired Student’s *t* test, and *p* value ≤ 0.05 * and 0.01 ** show grades of significance.

Similar to healthy individuals, we also explored the activation of lymphoid cells from baseline and SLGN-treated CHB subjects. A significant upregulation of the early activation marker CD69 was found on MAIT, CD8^+^ T and CD56^+^ NK cells, suggesting that a single dose of an oral selective-TLR8 agonist resulted in early activation of peripheral blood lymphoid cells in a subset of CHB patients similar to healthy donors (Figure 5A–F).

### 3.6. Selgantolimod (SLGN) Administration Modulated Global Gene Expression in Monocytes and Lymphocytes from PBMCs of Healthy Subjects

**Figure 6:**
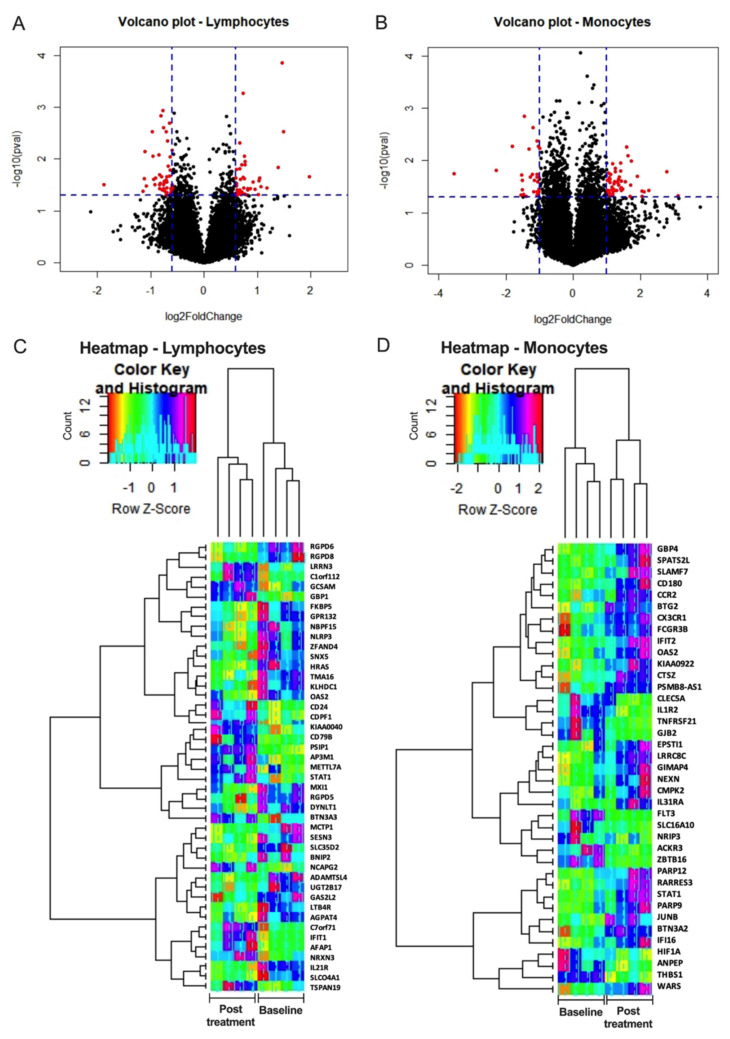
Differential gene expression analyses in lymphocytes and monocytes after oral-SLGN treatment in healthy subjects. Sorted monocytes and lymphocytes from BL and 8 h post-SLGN (3.0 mg) treatment were used for microarray analysis (*n* = 4). Volcano Plots show the transcriptomics analyses of differentially expressed mRNAs from (**A**) lymphocytes and (**B**) monocytes post-SLGN treatment versus pre-treatment (baseline) samples. Heatmap analysis of top 50% deregulated genes and dendrogram generated by cluster analysis in (**C**) lymphocytes and (**D**) monocytes are presented.

To discover immune pathways altered by activation of the TLR8 pathway, mRNA from sorted lymphocytes and monocytes was analyzed by microarray. Volcano plots and unsupervised hierarchical clustering showed the magnitude of alteration in the expression of mRNAs between baseline and 8 h post-treatment with SLGN (Figure 6A–D). In lymphocytes, 92 mRNAs (45 up and 47 down) were differentially expressed with SLGN treatment (log2fold change ≥1.5-fold, *p* < 0.05) (Figure 6A,C and Table S3). Differential expression analysis revealed 79 mRNAs (50 up and 29 down) were altered significantly in monocytes (log2fold change ≥2.0-fold, *p* < 0.05) upon SLGN treatment (Figure 6B,D and Table S2). Heatmaps generated with gene expression of the top 50% differentially expressed genes clearly identified the differences at baseline and post-SLGN treatment (Figure 6C,D).

**Figure 7:**
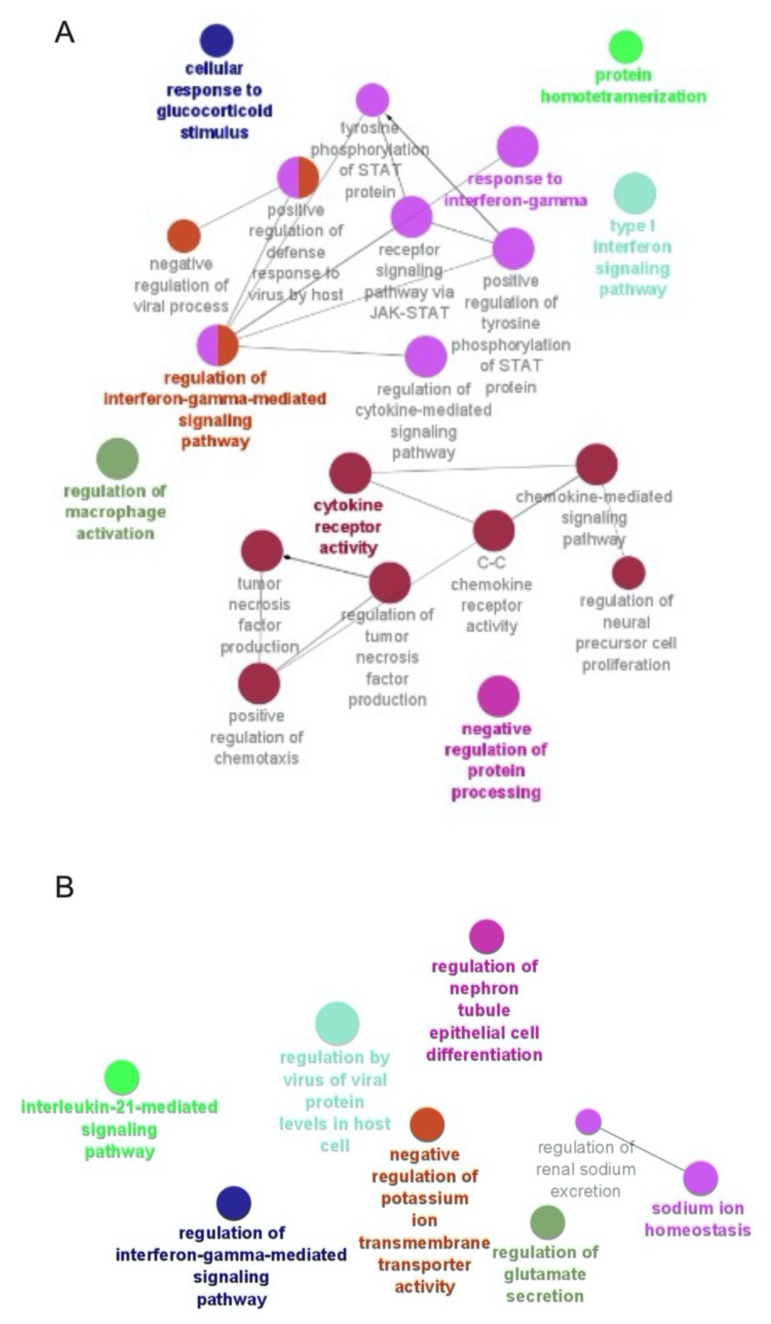
GO term enrichment analysis. The enriched functionally grouped networks of Biological Process category GO terms associated with deregulated genes from monocytes (**A**) and lymphocytes (**B**). The network of functionally grouped GO terms is built in Cytoscape v3.9.0 using the plugin ClueGO v2.5.8. The nodes are representative functional group GO terms and the edges are the associations based on a kappa score, as calculated in ClueGO. Different colors signify different GO terms’ functional groups. Only nodes with a *p*-value ≤ 0.05 are represented in the figures.

The functional enrichment of the differentially expressed genes (DEGs) is represented by a network of enriched functionally grouped GO terms of a biological process (BP) category, as depicted in Figure 7A,B. The BP GO terms associated with DEGs among monocytes were predominantly enriched in the regulation of macrophage activation (GO: 0043030), regulation of interferon-gamma-mediated signaling pathway (GO: 0060334), response to interferon-gamma (GO: 0034341), type-I interferon signaling pathway (GO: 0060337) and receptor signaling pathway via JAK-STAT (GO: 0007259) (Figure 7A). The BP GO terms associated with the DEGs among lymphocytes were predominantly enriched in the regulation of interferon-gamma-mediated signaling pathway (GO: 0060334), interleukin-21-mediated signaling pathway (GO: 0038114) and regulation by virus of viral protein in host cell (GO: 0046719) (Figure 7B).

The functional associations among the proteins of DEGs were tested based on co-expression evidence accessible to STRING (v11.5). The objective of this analysis is to find evidence of co-expression of some of the DEGs found in our analysis. Figure S3A,B represents the network of protein–protein interactions based on co-expression evidence among monocytes and lymphocytes, respectively. Some important interactions were clearly observed among monocytes, including proteins associated to interferon-gamma signaling (IFIT2, IFI16, OAS2, STAT1, JAK2, IRF2 and APOBEC3G) or monocyte activation and mobilization (FLT3 and CCR2). Similarly, evidence-based protein–protein interaction networks among lymphocytes, including proteins associated to interferon-gamma signaling (IFIT1, STAT1 and OAS2) and IL21- signaling (IL21R and STAT1), were observed. We observed only a few co-expression-based interactions among the DEGs in both monocytes and lymphocytes. A possible reason for this could be their involvement in different and un-related molecular pathways, or more studies need to be done to find the evidence of possible interactions among the deregulated genes. Although, most of the co-expression-based interactions we observed in the exploratory analysis with STRING had a moderate confidence score between 0.5–0.7 (Figure S3A,B); nonetheless, the presence of evidence of their interactions in literature is encouraging. Taken together, these results clearly illustrate that the DEGs altered some of the key molecular pathways, including cytokine receptor activity, chemokine receptor activity and interferon-gamma signaling pathways among the monocytes and lymphocytes, and they are linked to Selgantolimod treatment for 8 h, which may lead to activation of specific immune cell subsets. However, functional validations are needed to test these functional annotations.

## 4. Discussion

In this study, we show that a single oral administration of the selective TLR8 agonist SLGN induces activation of multiple innate and adaptive immune cells. SLGN administration resulted in activation of myeloid DCs and induced production of pro-inflammatory cytokines from these cells. Simultaneous activation of multiple effector lymphoid cells from innate (NK, MAIT) and adaptive (CD8^+^ T) arms occurred. Finally, increased effector potential of immune cells with SLGN administration was evident.

Immunomodulatory strategies that enhance immune effector function are under evaluation for the treatment of chronic viral infections, such as CHB [25]. Currently available nucleos(t)ide analogues effectively suppress HBV replication, however only 2–3% of people achieve the loss of a hepatitis B surface antigen (HBsAg), which defines a functional cure with these therapies [26]. This necessitates life-long therapy that comes with costs, side effects and potential for viral escape. In this regard, Toll-like receptor agonists are attractive immune stimulants being tested in combination therapy approaches that involve antiviral and immune modulatory components. SLGN is a selective TLR8 agonist developed for HBV functional cure approaches. Phase 1 studies have shown impressive activity of this molecule for enhancing antiviral immune responses [15,16,17]. Here we evaluated acute immune responses induced by a single oral administration of this agonist in healthy human subjects. A clear activation of myeloid cells (expression of co-stimulatory molecules CD40 and CD86) was evident, which was accompanied by pro-inflammatory cytokine production from mDCs. Previously, we showed that SLGN administration in healthy subjects induced dose-dependent pro-inflammatory cytokines IL-12p40, TNFα, IL-6, IL-10 and IL-1RA and a subset of chemokines CCL2 (MCP-1) and CXCL9 (MIG) in serum samples [17]. Mechanistically, we know from earlier in vitro work that IL-12 and IL-18 produced by monocytes upon TLR8 activation act on the cytokine receptors present on various lymphoid cells, including MAIT and NK cells, and enhance their effector function [9]. SLGN induced a similar effect in vivo, as demonstrated by upregulation of early activation marker CD69 on innate effector cells viz., MAIT and NK cells. A reduction in frequencies of these lymphoid cells was present in peripheral blood samples, which may indicate a migration of the cells to tissue compartments. We did not observe any significant changes in either apoptosis or proliferation markers on these cells upon SLGN administration (data not shown). Importantly, activated lymphoid cells expressed higher levels of antiviral cytotoxic molecules granzyme B and perforin. TLR8 signaling has the potential to suppress HBV replication via cytokines IFN-γ and TNF-α, as well as cytotoxic molecules perforin and granzyme B produced by cytotoxic lymphocytes [27]. Others have recently shown ex vivo stimulation with this agonist caused activation of innate and adaptive immune cells in peripheral blood samples from CHB patients [19]. Likewise, we performed in vitro stimulation of whole blood from healthy as well as CHB samples in tubes preloaded with this agonist (TruCulture) and observed activation of both myeloid and lymphoid cell populations, as well as induction of multiple cytokines (manuscript under preparation). In our study, a limited analysis of samples from a phase 1b trial with the same agonist showed comparable in vivo immune activation in CHB patients to that observed in healthy individuals.

A limitation of the study is the lack of integrated analysis of outcomes previously reported in our pharmacokinetic study and this study, though there is an indication of concordance between these data. For example, while both doses of SLGN induced immune changes, most effects were more pronounced, including some that were exclusively induced with higher dose of 3 mg, which agrees with dose-dependent cytokine induction with SLGN administration, as previously reported in a PK/PD study [17]. In continuation of this work, we are examining mechanisms of SLGN mediated immune changes in CHB clinical samples (phase 1b) as well in-depth integrated analyses of pharmacokinetic and immune changes.

Thus, SLGN has immunomodulatory activity in vivo in humans and activates multiple innate and adaptive immune cell responses. SLGN was tested in a phase 2 clinical trial in CHB patients, and analysis of those data is in progress. Our results also encourage further testing of this compound, with novel combination strategies aimed to achieve viral clearance as well as provide useful biomarkers to assess clinical responses with this agonist.

## Data Availability

The original data presented in the study are included in the article and Supplementary Materials. Further inquiries can be directed to the corresponding author.

## Supplementary Materials

The following are available online at www.mdpi.com/article/10.3390/v13122400/s1. Figure S1: Frequencies of peripheral blood immune cells after oral-SLGN administration in healthy subjects, Figure S2: Activation of myeloid and lymphoid cells after oral-SLGN treatment in healthy subjects, Figure S3: Protein-protein interaction networks, Table S1: List of flow cytometry antibodies, Table S2: List of genes deregulated in monocytes with 8 h Selgantolimod treatment and Table S3: List of genes deregulated in lymphocytes with 8 h Selgantolimod treatment.

## References

[1] Eigenbrod T., Pelka K., Latz E., Kreikemeyer B., Dalpke A.H. (2015). TLR8 Senses Bacterial RNA in Human Monocytes and Plays a Nonredundant Role for Recognition of Streptococcus pyogenes. J. Immunol..

[2] Gorden K.B., Gorski K.S., Gibson S.J., Kedl R.M., Kieper W.C., Qiu X., Tomai M.A., Alkan S.S., Vasilakos J.P. (2005). Synthetic TLR agonists reveal functional differences between human TLR7 and TLR8. J. Immunol..

[3] Jurk M., Heil F., Vollmer J., Schetter C., Krieg A.M., Wagner H., Lipford G., Bauer S. (2002). Human TLR7 or TLR8 independently confer responsiveness to the antiviral compound R-848. Nat. Immunol..

[4] Kawai T., Akira S. (2011). Toll-like receptors and their crosstalk with other innate receptors in infection and immunity. Immunity.

[5] Peng G., Guo Z., Kiniwa Y., Voo K.S., Peng W., Fu T., Wang D.Y., Li Y., Wang H.Y., Wang R.F. (2005). Toll-like receptor 8-mediated reversal of CD4+ regulatory T cell function. Science.

[6] Cervantes J.L., Weinerman B., Basole C., Salazar J.C. (2012). TLR8: The forgotten relative revindicated. Cell Mol. Immunol..

[7] Kieffer M.E., Patel A.M., Hollingsworth S.A., Seganish W.M. (2020). Small molecule agonists of toll-like receptors 7 and 8: A patent review 2014–2020. Expert Opin. Ther. Pat..

[8] Ayithan N., Tang L., Tan S.T., Chen D., Wallin J.J., Fletcher S.P., Kottilil S., Poonia B. (2021). Follicular helper T (TFH) cell targeting by TLR8 signaling for improving HBsAg-specific B cell response in chronic hepatitis B patients. Front. Immunol..

[9] Jo J., Tan A.T., Ussher J.E., Sandalova E., Tang X.Z., Tan-Garcia A., To N., Hong M., Chia A., Gill U.S. (2014). Toll-like receptor 8 agonist and bacteria trigger potent activation of innate immune cells in human liver. PLoS Pathog..

[10] Bennett M.S., Trivedi S., Iyer A.S., Hale J.S., Leung D.T. (2017). Human mucosal-associated invariant T (MAIT) cells possess capacity for B cell help. J. Leukoc. Biol..

[11] Ussher J.E., Willberg C.B., Klenerman P. (2018). MAIT cells and viruses. Immunol. Cell Biol..

[12] Alter G., Suscovich T.J., Teigen N., Meier A., Streeck H., Brander C., Altfeld M. (2007). Single-stranded RNA derived from HIV-1 serves as a potent activator of NK cells. J. Immunol..

[13] Schlaepfer E., Speck R.F. (2008). Anti-HIV activity mediated by natural killer and CD8+ cells after toll-like receptor 7/8 triggering. PLoS ONE.

[14] Giraldo D.M., Hernandez J.C., Urcuqui-Inchima S. (2016). HIV-1-derived single-stranded RNA acts as activator of human neutrophils. Immunol. Res..

[15] Mackman R.L., Mish M., Chin G., Perry J.K., Appleby T., Aktoudianakis V., Metobo S., Pyun P., Niu C., Daffis S. (2020). Discovery of GS-9688 (Selgantolimod) as a Potent and Selective Oral Toll-Like Receptor 8 Agonist for the Treatment of Chronic Hepatitis, B. J. Med. Chem..

[16] Daffis S., Balsitis S., Chamberlain J., Zheng J., Santos R., Rowe W., Ramakrishnan D., Pattabiraman D., Spurlock S., Chu R. (2020). Toll-Like Receptor 8 Agonist GS-9688 Induces Sustained Efficacy in the Woodchuck Model of Chronic Hepatitis, B. Hepatology.

[17] Reyes M., Lutz J.D., Lau A.H., Gaggar A., Grant E.P., Joshi A., Mackman R.L., Ling J., Tan S.K., Ayithan A. (2020). Safety, pharmacokinetics and pharmacodynamics of selgantolimod, an oral Toll-like receptor 8 agonist: A Phase Ia study in healthy subjects. Antivir. Ther..

[18] Gane E.J., Kim H.J., Visvanathan K., Kim Y.J., Nguyen A.H., Wallin J.J., Chen D.Y., McDonald C., ARORA p., Tan S.K. (2021). Safety, pharmacokinetics, and pharmacodynamics of the oral TLR8 agonist selgantolimod in chronic hepatitis B. Hepatology.

[19] Amin O.E., Colbeck E.J., Daffis S., Khan S., Ramakrishnan D., Pattabiraman D., Chu R., Steuer H.M., Lehar S., Peiser L. (2021). Therapeutic potential of TLR8 agonist GS-9688 (selgantolimod) in chronic hepatitis B: Re-Modelling of antiviral and regulatory mediators. Hepatology.

[20] Bindea G., Mlecnik B., Hackl H., Charoentong P., Tosolini M., Kirilovsky A., Fridman W.-H., Pagès F., Trajanoski Z., Galon J. (2009). ClueGO: A cytoscape plug-in to decipher functionally grouped gene ontology and pathway annotation networks. Bioinformatics.

[21] Jensen L.J., Kuhn M., Stark M., Chaffron S., Creevey C., Muller J., Doerks T., Julien P., Roth A., Simonovic M. (2009). STRING 8—A global view on proteins and their functional interactions in 630 organisms. Nucleic Acids Res..

[22] Ussher J.E., Bilton M., Attwod E., Shadwell J., Richardson R., Lara d.C., Mettke E., Kurioka A., Hansen T.H., Klenerman P. (2014). CD161++ CD8+ T cells, including the MAIT cell subset, are specifically activated by IL-12+IL-18 in a TCR-independent manner. Eur. J. Immunol..

[23] Wilgenburg v.B., Scherwitzl I., Hutchinson E.C., Leng T., Kurioka A., Kulicke C., Lara C.d., Cole S., Vasanawathana S., Limpitikul W. (2016). MAIT cells are activated during human viral infections. Nat. Commun..

[24] Boeijen L.L., Montanari N.R., Groen d.R.A., Oord d.G.W., Heide-Mulder M.v.d., Knegt R.J.d., Boonstra A. (2017). Mucosal-Associated Invariant T Cells Are More Activated in Chronic Hepatitis B, but Not Depleted in Blood: Reversal by Antiviral Therapy. J. Infect. Dis..

[25] Locy H., Mey S.d., Mey W.d., Ridder M.D., Thielemans K., Maenhout S.K. (2018). Immunomodulation of the Tumor Microenvironment: Turn Foe into Friend. Front. Immunol..

[26] Ma Z., Cao Q., Xiong Y., Zhang E., Lu M. (2018). Interaction between Hepatitis B Virus and Toll-Like Receptors: Current Status and Potential Therapeutic Use for Chronic Hepatitis, B. Vaccines.

[27] Deng G., Ge J., Liu C., Pang J., Huang Z., Peng J., Sun J., Hou J.L., Zhang X.Y. (2017). Impaired expression and function of TLR8 in chronic HBV infection and its association with treatment responses during peg-IFN-alpha-2a antiviral therapy. Clin. Res. Hepatol. Gastroenterol..

