# Supplementary material for: Oral Selective TLR8 Agonist Selgantolimod Induces Multiple Immune Cell Responses in Humans
Source: Viruses. 2021 Nov 30;13(12):2400. doi: 10.3390/v13122400 (PMC8706304; doi:10.3390/v13122400)
Supplement: Supplementary file 1 [file viruses-13-02400-s001.zip › viruses-1469764-supplementary.pdf]

viruses

MDPI

Supplementary Materials

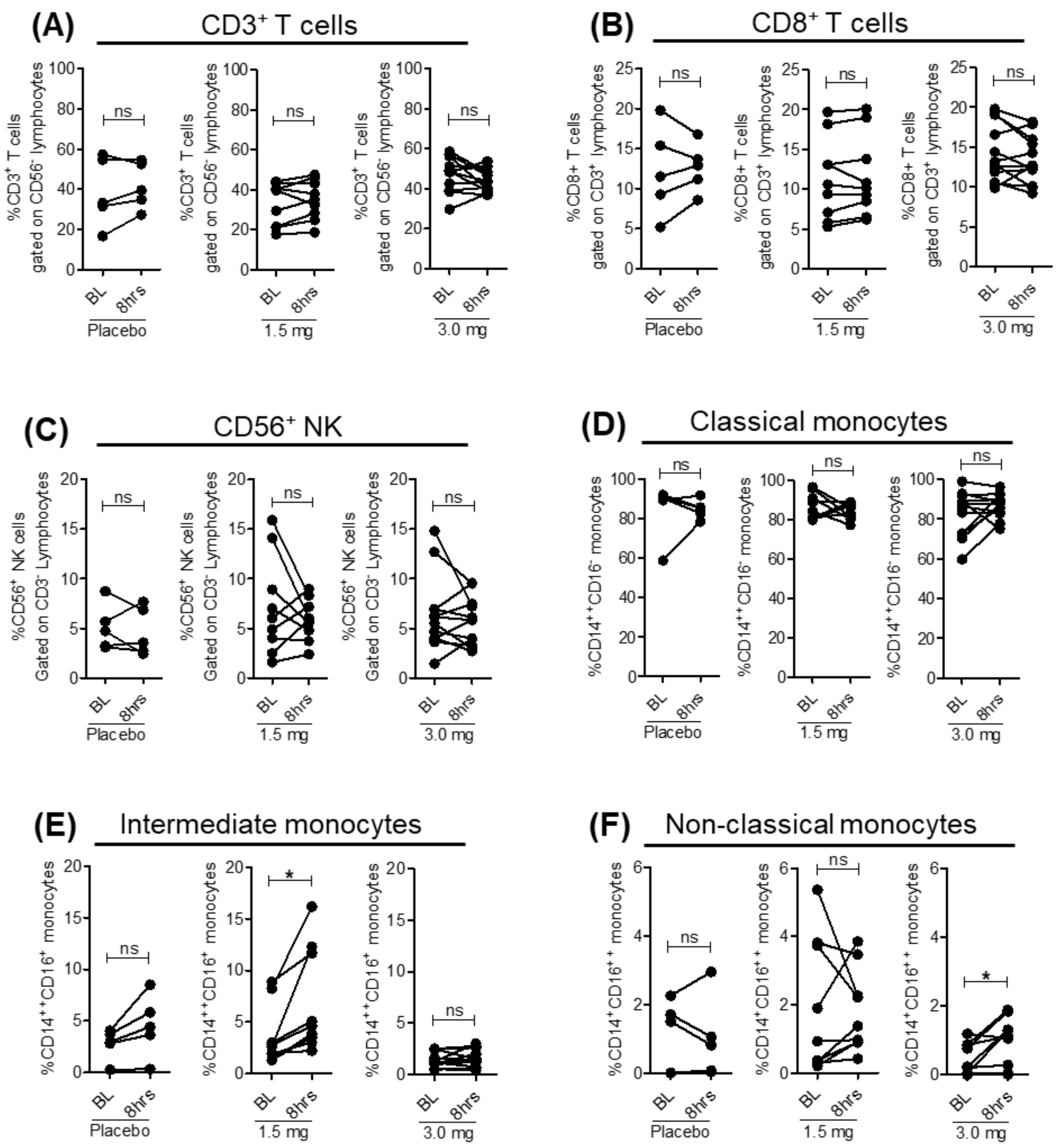

Figure S1. Frequencies of peripheral blood immune cells after oral-SLGN administration in healthy subjects. Flow cytometric analysis of PBMCs from BL (Pre) and 8hrs post-treatment) from individuals administered placebo (n=5) or indicated dosage of SLGN (1.5 mg (n=9) and 3.0 mg (n=12)). Comparative analyses of frequencies of immune cell subsets from baseline and 8-hour samples in placebo or SLGN-treated individuals for (**A**) $CD3^+$ T, (**B**) $CD8^+$ T, (**C**) $CD56^+$ NK and monocyte subsets (**D**) $CD14^{++}CD16^-$ classical monocytes, (**E**) $CD14^{++}CD16^+$ intermediate monocytes and (**F**) $CD14^+CD16^{++}$ non-classical monocytes are shown. Significance calculated by Wilcoxon matched-pairs signed rank test . P values ≤0.05* indicates the statistical significance. ns; no significance.

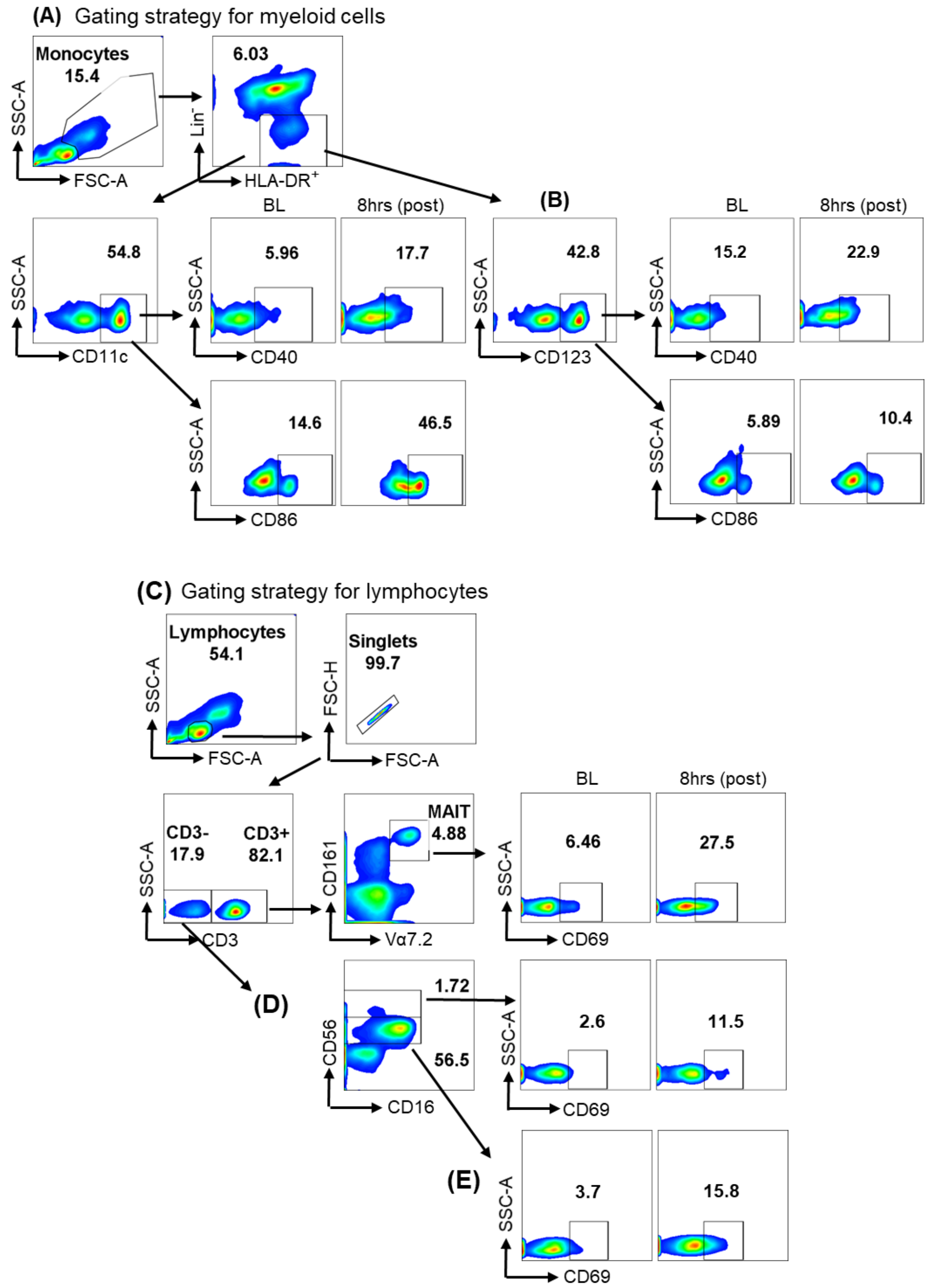

Figure S2. Activation of myeloid and lymphoid cells after oral-SLGN treatment in healthy subjects. (**A-B**) Gating strategy for myeloid cells shows the expression of CD40 and CD86 on Lin$^-$HLA-DR$^+$CD11c$^+$ mDCs and Lin$^-$HLA-DR$^+$CD123$^+$ pDCs respectively from baseline and 8hrs post-SLGN (3.0 mg) treated individuals. (**C-E**) Gating strategy for lymphocytes displays the cell surface expression of CD69 on MAIT, CD56^{bright} and CD56^{dim} NK cell subsets. BL; baseline.

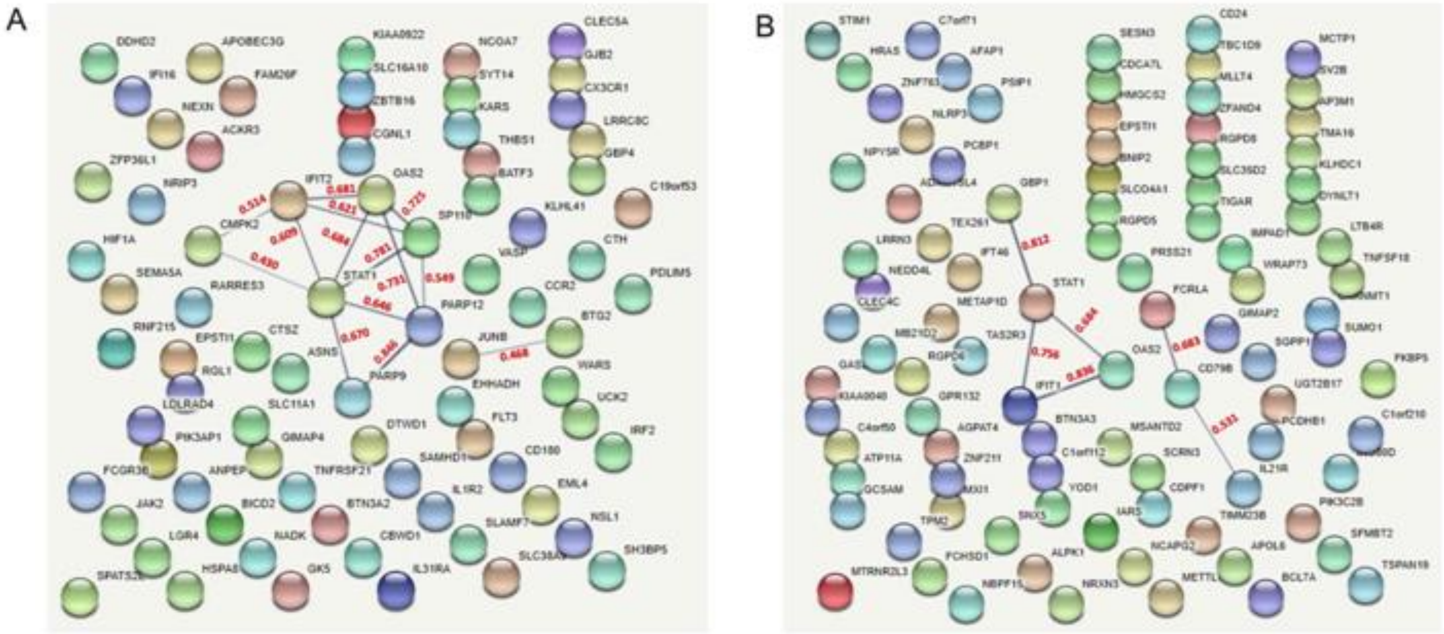

Figure S3. Protein-protein interaction networks were generated using STRING online tool (string-db.org) with medium confidence score >= 0.4. (**A**) Interaction network based on co-expression evidence with DEGs among monocytes. (**B**) Interaction network based on co-expression evidence with DEGs among lymphocytes. The interaction network is created with nodes as the gene/protein and edges as significant evidence from publicly available co-expression data accessible to STRING. The width and the numbers associated with the edges represents the confidence scores of the co-expression-based interactions.

Table S1: List of flow cytometry antibodies

Sl. No.	Flow Panel	Markers	Fluorochrome	Clone	Company	Cat. #
1	**MAIT**	CD69	FITC	FN50	Biolegend	310904
2		CD161	PE	191B8	Miltenyi Biotec	130-113-593
3		CD38	PerCp/Cy5.5	HIT2	Biolegend	303522
4		Vα7.2	PE/Cy7	3C10	Biolegend	351712
5		Granzyme B	APC	QA16A02	Biolegend	372204
6		CD8a	BV605	RPA-T8	Biolegend	301040
7		CD3	APC/Cy7	OKT3	Biolegend	317342
8		Live/Dead Aqua	APC/Cy7	––	Thermo Fisher	L34965
9	**NK**	Perforin	FITC	B-D48	Biolegend	353310
10		CD16	PE/Cy7	3G8	Biolegend	302016
11		Granzyme B	BV421	GB11	BD Biosciences	563389
12		CD56	BV510	HCD56	Biolegend	318340
13		CD69	BV605	FN50	Biolegend	310938
14	**Cytokines**	IL-1β	FITC	JK1B-1	Biolegend	508206
15		IL-18	PE	74801	R&D Systems	IC646P
16		IL-12/IL-23 p40	PerCp/Cy5.5	C11.5	Biolegend	501822
17		CD14	BV421	M5E2	Biolegend	301830
18		IL-6	APC	MQ2-13A5	Biolegend	501112
19		TNFα	BV510	MAb11	Biolegend	502950
20	**mDCs / pDCs**	CD11c	BV605	3.9	Biolegend	301636
21		Lineage Cocktail (CD3/14/16/19/20/56)	FITC	UCHT1/HCD14/3G8/HIB19/2H7/HCD56	Biolegend	348801
22		CD123	PE	6H6	Biolegend	306006
23		CD86	BV421	IT2.2	Biolegend	305426
24		HLA-DR	BV510	L243	Biolegend	307646
25		CD40	APC/Cy7	5C3	Biolegend	334324
26	**Monocytes**	HLA-DR	PE	L243	Biolegend	307606
27		CD14	PerCp/Cy5.5	63D3	Biolegend	367110
28		CD16	BV605	3G8	Biolegend	302040

Table S2: List of genes deregulated in monocytes with 8 hr Selgantolimod treatment

Gene symbols	Mean exp (post-treatment, log2)	Variance (post-treatment)	Mean exp (pre-treatment, log2)	Variance (pre-treatment)	Fold Change (Log2)	p-value	Probe_IDs
IFIT2	11.54	3.02	8.42	0.6	3.12	0.04725	NM_001547
CX3CR1	11.31	0.54	8.53	2.64	2.78	0.0165	NM_001171171
OAS2	10.71	1.22	8.48	1.53	2.24	0.03715	NM_001032731
WARS	15.84	0.72	13.73	0.9	2.11	0.03929	NM_004184
GIMAP4	9.44	1.26	7.38	0.94	2.06	0.03786	NM_018326
FCGR3B	10.7	0.31	8.64	2.85	2.05	0.04865	NM_000570
PARP9	12.7	1.65	10.8	0.98	1.91	0.01987	NM_001146102
GBP4	10.59	0.47	8.86	0.18	1.73	0.01029	NM_052941
BTG2	10.79	0.23	9.08	0.55	1.72	0.0297	NM_006763
RARRES3	13.13	0.58	11.45	0.31	1.68	0.04772	NM_004585
CD180	10.96	0.86	9.34	0.23	1.62	0.00802	NM_005582
JUNB	13.63	0.5	12.03	0.28	1.6	0.00541	NM_002229
BTN3A2	13.48	0.08	11.93	0.57	1.55	0.02544	NM_001197246
LRRC8C	9.09	0.32	7.62	0.65	1.47	0.03472	NM_032270
PSMB8-AS1	11.83	0.02	10.37	0.62	1.46	0.03375	NR_037173
PARP12	13.01	0.57	11.58	0.31	1.44	0.02439	NM_022750
KIAA0922	11.34	0.39	9.91	0.28	1.43	0.03744	NM_001131007
SLAMF7	10.14	0.81	8.71	0.11	1.43	0.03279	NM_001282588
IRF2	11.33	0.55	9.93	0.06	1.41	0.01101	NM_002199
IFI16	14.09	0.83	12.69	0.63	1.4	0.01569	NM_001206567
STAT1	12.47	0.8	11.07	0.35	1.4	0.03358	NM_007315
CTSZ	11.26	0.29	9.88	0.46	1.37	0.02049	NM_001336
SP110	11.63	0.48	10.27	0.18	1.36	0.0291	NM_001185015
NEXN	8.47	1.21	7.13	0.51	1.34	0.02245	NM_001172309
VASP	13.27	0.05	11.93	0.45	1.33	0.02711	NM_003370
SAMHD1	12.51	0.21	11.19	0.42	1.32	0.02501	NM_015474
SPATS2L	10.2	1.05	8.9	0.14	1.3	0.03679	NM_001100422
DTWD1	8.2	0.09	6.93	0.25	1.27	0.03705	NM_001144955
HSPA8	17.59	0.44	16.33	0.36	1.26	0.01394	NM_006597
JAK2	13.03	0.55	11.77	0.11	1.26	0.01623	NM_004972
APOBEC3G	8.5	0.45	7.27	0.08	1.23	0.02541	NM_021822
IL31RA	7.76	1.46	6.54	0.41	1.22	0.02785	NM_001242636
EML4	11.11	0.04	9.91	0.59	1.2	0.0381	NM_001145076
NADK	11.61	0.04	10.43	0.46	1.18	0.04561	NM_001198993
EPSTI1	9.08	0.72	7.9	1.12	1.18	0.04239	NM_001002264
CMPK2	9.53	1.6	8.35	1.09	1.18	0.01152	NM_001256477
CCR2	10.4	1.06	9.22	0.89	1.18	0.0347	NM_001123041
CBWD1	13.74	0.15	12.58	0.14	1.16	0.03609	NM_001145355
NSL1	8.46	0.05	7.31	0.49	1.15	0.03248	NM_001042549
FAM26F	7.6	0.2	6.51	0.1	1.1	0.04689	NM_001010919
ZFP36L1	14.47	0.46	13.37	0.63	1.1	0.01825	NM_001244698
BATF3	8.99	0.11	7.9	0.04	1.09	0.02586	NM_018664
SLC38A9	6.08	0.11	4.98	0.21	1.09	0.02241	NM_001258286
RGL1	9.44	0.38	8.38	0.23	1.07	0.02096	NM_001297669
BICD2	7.21	0.05	6.14	0.21	1.07	0.0408	NM_001003800
NCOA7	9.13	0.4	8.07	0.2	1.06	0.01707	NM_001122842
C19orf53	10.08	0.06	9.03	0.58	1.04	0.03758	NM_014047

PDLIM5	9.15	0.12	8.12	0.27	1.03	0.04112	NM_001011513
KARS	12.72	0.3	11.7	0.13	1.02	0.02419	NM_001130089
PIK3AP1	13.5	0.12	12.48	0.57	1.02	0.03716	NM_152309
UCK2	5.27	0.11	6.28	0.01	-1	0.00577	hsa_circ_0007260
LGR4	6.32	0.23	7.33	0.01	-1.01	0.03915	NM_018490
LDLRAD4	10.36	0.28	11.39	0.35	-1.02	0.02139	NM_001003674
MIR670HG	5.11	0.14	6.14	0.31	-1.03	0.0053	NR_131246
EHHADH	6.85	0.15	7.87	0.15	-1.03	0.0355	NM_001166415
DDHD2	6	0.45	7.04	0.06	-1.04	0.03829	NM_001164232
CGNL1	4.68	0.1	5.72	0.37	-1.04	0.0413	NM_001252335
SH3BP5	9.38	0.62	10.45	0.44	-1.07	0.00427	NM_001018009
CTH	6.72	0.21	7.79	0.43	-1.07	0.02525	NM_001190463
NRIP3	6.81	1.05	7.89	1.83	-1.08	0.03981	NM_020645
SEMA5A	5.32	0.08	6.41	0.1	-1.09	0.01816	NM_003966
LINC01089	8.75	0.32	9.83	0.01	-1.09	0.04524	NR_002809
RNF215	4.68	0.05	5.77	0.09	-1.09	0.02051	ENST00000619645
SLC11A1	13.23	0.28	14.38	0.01	-1.15	0.02365	NM_000578
KLHL41	4.07	0.12	5.27	0.33	-1.19	0.00237	NM_006063
ASNS	5.79	0.07	6.98	0.36	-1.19	0.03893	NM_001178075
SYT14	5.45	0.21	6.67	0.02	-1.22	0.01812	NM_001146261
HIF1A	14.98	0.66	16.26	1	-1.28	0.02072	NM_001243084
ANPEP	14.32	0.27	15.64	0.59	-1.32	0.00596	NM_001150
SLC16A10	6.11	0.13	7.51	1.18	-1.4	0.0488	NM_018593
CLEC5A	9.37	1.35	10.82	1.25	-1.45	0.00144	NM_001301167
FLT3	6.46	0	7.93	0.74	-1.47	0.04375	NM_004119
GK5	9.32	0.19	10.84	0.17	-1.52	0.02377	NM_001039547
ACKR3	7.31	0.1	8.83	0.94	-1.52	0.03567	NM_020311
IL1R2	8.65	0.47	10.18	0.57	-1.53	0.0189	NM_001261419
GJB2	7.97	0.15	9.53	1.36	-1.57	0.04573	NM_004004
ZBTB16	7.11	0.06	8.92	0.43	-1.82	0.00536	NM_001018011
TNFRSF21	7.79	0.2	10.08	1.31	-2.3	0.01518	NM_014452
THBS1	13.39	1.58	16.94	0.07	-3.55	0.01783	NM_003246

Table S3: List of genes deregulated in lymphocytes with 8 hr Selgantolimod treatment

Gene symbols	Mean exp (pre-treatment, log2)	Variance (pre-treatment)	Mean exp (post-treatment, log2)	Variance (post-treatment)	Fold Change (Log2)	p-value	Probe_IDs
STAT1	9.69	0.89	11.66	0.76	1.98	0.02209	NM_007315
GBP1	7.86	0.17	9.34	0.21	1.49	0.00297	NM_002053
PSIP1	9.56	0.04	11.03	0.07	1.47	0.00014	NM_001128217
KIAA0040	9.82	0.42	11.21	0.15	1.39	0.01453	NM_001162893
LRRN3	9.09	0.45	10.27	0.31	1.18	0.03635	NM_001099658
OAS2	7.43	0.09	8.5	0.39	1.07	0.03384	NM_001032731
IFIT1	5.67	0.1	6.72	0.31	1.05	0.02332	NM_001270927
C7orf71	5.76	0.25	6.79	0.24	1.03	0.02632	NM_001145531
CD79B	9.96	0.05	10.97	0.44	1.02	0.04874	NM_000626
GCSAM	8.19	0.34	9.15	0.07	0.96	0.03838	NM_001190259
NRXN3	5.31	0.19	6.22	0.21	0.91	0.02754	NM_001105250
BTN3A3	11.43	0.27	12.3	0.12	0.87	0.03734	NM_001242803
AFAP1	5.42	0.04	6.24	0.24	0.82	0.03716	NM_001134647
AP3M1	10.59	0.15	11.41	0.23	0.82	0.03915	NM_012095
C1orf112	8.66	0.1	9.44	0.26	0.78	0.04808	NM_018186
NCAPG2	6.43	0.06	7.2	0.12	0.77	0.01298	NM_001281932
FCRLA	10.56	0.03	11.31	0.09	0.76	0.0087	NM_001184866
MSANTD2	6.96	0	7.72	0.13	0.76	0.02364	NM_001308027
PCBP1	11.77	0.11	12.54	0.05	0.76	0.01121	NM_006196
METTL7A	10.47	0.23	11.22	0.12	0.75	0.04724	NM_014033
CARNMT1	10.22	0.15	10.95	0.04	0.74	0.02275	NM_152420
CD24	7.32	0.1	8.06	0.19	0.74	0.03577	NM_001291737
SGPP1	6.37	0.03	7.1	0.02	0.73	0.00054	NM_030791
TSPAN19	4.66	0.07	5.37	0.2	0.71	0.04332	NM_001100917
STIM1	9.89	0.08	10.59	0	0.7	0.01297	NM_001277961
CDPF1	7.13	0.21	7.82	0.05	0.69	0.04827	NM_207327
NPY5R	6.31	0.01	7	0.12	0.68	0.0245	NM_006174
EPSTI1	7.27	0.18	7.93	0.06	0.67	0.04237	NM_001002264
NEDD4L	6.7	0.09	7.38	0.07	0.67	0.01549	NM_001144964
MB21D2	6.3	0.11	6.98	0.15	0.67	0.03899	NM_178496
TBC1D9	6.51	0.05	7.18	0.05	0.67	0.0049	NM_015130
APOL6	7.45	0.04	8.08	0.16	0.63	0.04155	NM_030641
MLLT4	5.76	0.06	6.4	0.17	0.63	0.04769	NM_001040000
METAP1D	6.35	0.13	6.97	0.12	0.62	0.04911	NM_199227
C1orf210	8.02	0.14	8.64	0.1	0.62	0.04715	NM_001164829
YOD1	6.5	0.07	7.11	0.11	0.61	0.02898	NM_001276320
IFT46	7.48	0.06	8.08	0.07	0.61	0.0157	NM_001168618
SUMO1	10.76	0.04	11.37	0.12	0.61	0.0301	NM_001005781
HMGCS2	4.55	0.05	5.16	0.12	0.61	0.03136	NM_001166107
TAS2R3	5.51	0.13	6.12	0.11	0.61	0.04941	NM_016943
CDCA7L	9.37	0.14	9.99	0.05	0.61	0.03924	NM_001127370
BCL7A	5.71	0.01	6.31	0.09	0.6	0.02313	NM_001024808
PIK3C2B	6.74	0.1	7.34	0.09	0.6	0.03252	NM_002646
SV2B	6.11	0.13	6.7	0.08	0.59	0.04549	NM_001167580
PCDHB1	5.8	0.08	6.4	0.15	0.59	0.04862	NM_013340
TIGAR	6.83	0.13	6.23	0.05	-0.59	0.03623	NM_020375
TIMM23B	11.99	0.05	11.39	0.03	-0.6	0.00678	NM_001290117
SCRN3	7.48	0.1	6.88	0.08	-0.6	0.03145	NM_001193528

ALPK1	6.74	0.12	6.14	0.03	-0.6	0.03387	NM_001102406
C4orf50	7.4	0.15	6.79	0.06	-0.61	0.04194	BC140710
SFMBT2	8.84	0.12	8.21	0.12	-0.62	0.04449	NM_001018039
TEX261	9.62	0.08	8.99	0.13	-0.62	0.0372	NM_144582
WRAP73	5.97	0.05	5.34	0.06	-0.63	0.00917	NM_017818
INO80D	8.96	0.09	8.32	0.04	-0.64	0.01626	NM_017759
PRSS21	6.83	0.01	6.18	0.09	-0.65	0.02092	NM_001270452
MTRNR2L3	7.72	0.03	7.07	0.03	-0.65	0.00202	NM_001190472
TNFSF18	5.53	0.09	4.86	0.05	-0.67	0.01384	NM_005092
ATP11A	7.02	0.04	6.32	0.17	-0.69	0.03621	NM_015205
TRPC5OS	5.39	0.05	4.7	0.05	-0.69	0.00443	NM_001195576
IMPAD1	8.59	0.15	7.88	0.04	-0.7	0.02756	NM_017813
ZNF763	8.33	0.19	7.63	0.1	-0.7	0.04413	NM_001012753
NBPF15	9.28	0.15	8.59	0.17	-0.7	0.04912	NM_001170755
TPM2	7.48	0.13	6.79	0.04	-0.7	0.02175	NM_001301226
GIMAP2	8.25	0.03	7.54	0.14	-0.71	0.02556	NM_015660
BNIP2	7.03	0.22	6.33	0.06	-0.71	0.0479	NM_004330
FCHSD1	9.98	0.05	9.26	0.03	-0.72	0.00295	NM_033449
SLC35D2	7.24	0.22	6.5	0.06	-0.74	0.04229	NM_001286990
MXI1	11.01	0.16	10.26	0.18	-0.75	0.04074	NM_001008541
GAS2L2	6.26	0.08	5.51	0.16	-0.75	0.02658	NM_139285
ADAMTSL4	6.78	0.18	6.03	0.05	-0.75	0.0288	NM_001288607
SLCO4A1	5.59	0.19	4.82	0.04	-0.76	0.03118	NM_016354
UGT2B17	6.46	0.19	5.7	0.13	-0.76	0.03713	NM_001077
CLEC4C	7.87	0.05	7.11	0.03	-0.76	0.00246	NM_130441
ZNF211	9.12	0.03	8.36	0.04	-0.77	0.00117	NM_001265597
MCTP1	7.45	0.23	6.67	0.04	-0.78	0.0395	NM_001002796
RGPD5	10.91	0.05	10.11	0.17	-0.79	0.02203	NM_005054
IARS	9.27	0	8.46	0.03	-0.81	0.00147	NM_002161
RGPD8	9.98	0.22	9.16	0.06	-0.82	0.02954	NM_001164463
KLHDC1	7.75	0.15	6.92	0.23	-0.83	0.03751	NM_172193
ZFAND4	8.39	0.14	7.55	0.21	-0.84	0.03099	NM_001128324
SESN3	7.39	0.17	6.55	0.1	-0.84	0.02022	NM_001271594
HRAS	7.87	0.09	7.02	0.11	-0.85	0.0083	NM_005343
TMA16	7.79	0.19	6.87	0.24	-0.92	0.03082	NM_018352
LTB4R	6.94	0.23	6.02	0.11	-0.92	0.02352	NM_001143919
AGPAT4	6.93	0.19	5.99	0.21	-0.94	0.02488	NM_020133
SNX5	8.08	0.15	7.12	0.04	-0.96	0.00869	hsa_circ_0008446
IL21R	5.85	0.08	4.88	0.09	-0.97	0.003	NM_021798
FKBP5	9.79	0.38	8.72	0.21	-1.08	0.03332	NM_001145775
RGPD6	10.15	0.18	9.03	0.12	-1.12	0.00718	NM_001037866
DYNLT1	11.99	0.21	10.86	0.33	-1.13	0.0236	NM_001291602
NLRP3	9.01	0.44	7.87	0.34	-1.15	0.04124	NM_001079821
GPR132	10.01	0.92	8.13	0.9	-1.88	0.03138	NM_001278694
